# Sodium alginate potentiates antioxidant defense and PR proteins against early blight disease caused by *Alternaria solani* in *Solanum lycopersicum* Linn.

**DOI:** 10.1371/journal.pone.0223216

**Published:** 2019-09-30

**Authors:** Priya Dey, Ramani Ramanujam, Ganesan Venkatesan, Radhakrishnan Nagarathnam

**Affiliations:** 1 Unit of Plant Pathology, Centre for Advance Studies in Botany, University of Madras, Guindy Campus, Chennai, Tamil Nadu, India; 2 Acme Progen Biotech (India) Pvt. Ltd, Salem, Tamil Nadu, India; National Institutes of Health, UNITED STATES

## Abstract

The use of biopolymers as elicitors in controlling plant diseases is gaining momentum world-wide due to their eco-friendly and non-toxic nature. In the present study, we have used an algal biopolymer (sodium alginate) and tested its applicability as an elicitor in inducing resistance factors against *Alternaria solani*, which causes early blight disease in *Solanum lycopersicum* (tomato plant). We have pre-treated tomato plants with different concentrations of sodium alginate (0.2%, 0.4%, and 0.6%) before *A*. *solani* infection. We found that sodium alginate has effectively controlled the growth of *A*. *solani*. In addition, a significant increase in the expression levels of SOD was observed in response to pathogen infection. The increased protease inhibitors activity further suggest that sodium alginate restrict the development of *A*. *solani* infection symptoms in tomato leaves. This corroborates well with the cell death analysis wherein increased sodium alginate pre-treatment results in decreased cell death. Also, the expression profile analyses reveal the induction of genes only in sodium alginate-pretreated tomato leaves, which are implicated in plant defense mechanism. Taken together, our results suggest that sodium alginate can be used as an elicitor to induce resistance against *A*. *solani* in tomato plants.

## Introduction

Plants have evolved with a variety of defense mechanisms to resist pathogen invasion, e.g., the activation of systemic acquired resistance (SAR) that protects the host plant against a wide range of pathogens [1,2]. The key regulator of SAR in plants is salicylic acid (SA), which induces the expression of specific antimicrobial proteins [3]. The onset of SAR is manifested by the accumulation of novel proteins called pathogenesis-related proteins that are expressed by the host plants in response to pathological or related situations. They have been found to enhance the defensive capacity of plants in response to necrotic infections [4].

The primary defense response in the host plant against a pathogen involves the rapid generation of reactive oxygen species (ROS), also known as oxidative burst. ROS include reduced and chemically reactive molecules, such as superoxide anion (O_2_^−^), hydrogen peroxide (H_2_O_2_), hydroxyl radical (OH^•^), and hydroperoxyl radical (HO_2_^•^) [5]. ROS play important roles during the early stages of pathogen infection, which involve direct antimicrobial action, lignin formation, phytoalexin production, and SAR onset [6]. The balance between ROS production and antioxidation is important for maintaining a healthy biological system [7]. To mitigate the cell damage caused by ROS, plants express enzymes that scavenge excess ROS produced in cells under stressed conditions. These enzymes include superoxide dismutase (SOD; EC 1.15.1.1), peroxidase (POX, EC 1.11.1.7), ascorbate peroxidase (EC 1.11.1.11), and catalase (CAT; EC 1.11.1.6) [8]. SOD is the major antioxidant enzyme that catalyzes the dismutation of O_2_^−^ into H_2_O_2_ and oxygen (O_2_) [9]. In turn, CAT and POXs, such as guaiacol peroxidase (GPX), break down H_2_O_2_ into water (H_2_O) and O_2_ in the living cells [10,11]. There are mainly three types of SOD (Fe-SOD, Mn-SOD, Cu/Zn-SOD) present in plants and they are classified based on the metal cofactor present at the active site of the enzyme. They can also be distinguished by exploiting their sensitivity to cyanide (KCN) and hydrogen peroxide [12].

Defense signaling in plants can be induced using elicitors—molecules that help in inducing the defense responses in plants [13,14]. Elicitors are thought to interact with the major signaling molecules of the plant defense pathway, such as SA and jasmonic acid (JA)/ethylene (ET), thereby triggering the expression of SAR and induced systemic resistance, respectively [15].

Tomato (*Solanum lycopersicum* L., syn. *Lycopersicon esculentum* Miller) belongs to the Solanaceae family and is the second most widely used vegetable in the world after potato because of its high nutritional value and antioxidative properties [16]. Tomato is a well-characterized model plant system because of its relatively small genome size of 950 mb, diploid genome with 12 chromosome pairs, and short generation time, all of which make the plant suitable for genetic analysis [17]. One of the most devastating diseases of tomato is early blight caused by the necrotrophic fungi *Alternaria solani* (Ellis and Martin). A key approach to control this disease is by spraying fungicides. However, fungicides harm our environment by causing water and soil pollution and negatively affect soil microorganisms and animal and human health. These limitations could be overcome by adopting alternative strategies such as using certain microbes and biopolymers as potent elicitors for priming of the plant defense armory to vanquish the pathogen invasion [18,19].

Algal polysaccharides are one of the most abundant organic molecules thought of having a great molecular biodiversity that has not yet been completely elucidated. Over the last decades, these macromolecules have been found to possess enormous potential as elicitors of plant defense responses [20–22]. Sodium alginate, the sodium salt of alginic acid, is a biopolysaccharide. Alginic acid is a linear 1,4-linked copolymer of β-D-mannuronic acid and α-L-guluronic acid that can be arranged in heteropolymeric and homopolymeric blocks [23]. The major natural sources of alginates are seaweeds (class Phaeophyceae) such as *Macrocystis pyrifera*, *Ascophyllum nodosum*, *Sargassum sinicola* and various types of *Laminaria*. In the last decades, researchers have reported that alginate-derived oligosaccharides enhance seed germination, shoot elongation, and root growth [24,25]. Küpper et al. examined the activity of alginate oligomers and found that oligoguluronate is the most active fraction that elicits oxidative burst in kelp sporophyte (*Laminaria digitata*) [26]. Although alginate exhibits elicitor-like activity in plants, it is yet to be known whether it induces antioxidant defense responses in plants. Hence, this study aimed to examine the potential of biopolymer sodium alginate to induce resistance factors against *A*. *solani*-caused early blight disease in tomato plants.

## Materials and methods

### Plant materials and growth conditions

Seeds of tomato (*S*. *lycopersicum* Linn.) variety PKM1 (Periyakulam-1) were obtained from Tamil Nadu Horticulture Research Station, Periyakulam, Tamil Nadu, India. Tomato plants were grown in pots containing vermiculite soil for 35 days in a greenhouse under 16-/8-h light/dark cycle at 25°C. After 35 days, the plants were subjected to elicitor treatment and pathogen infection.

### Fungal culture

*A*. *solani* (Ellis and Martin) Sorauer (Accession No. 7114) culture was obtained from Indian Agricultural Research Institute, New Delhi, India and was grown on potato dextrose Agar media for 2 days. Subsequently, fungal mycelial blocks were cut from the media and transferred onto the surface of corn meal agar media (1.5% corn meal, 1.5% sucrose, and 2% agar). The culture was then incubated in dark at 25°C for 8 days.

### Elicitor treatment

Sodium alginate (Loba Chemie, India) was used as the elicitor. Spray solutions of different sodium alginate concentrations (0.2%, 0.4%, and 0.6%) were prepared using 0.02% (v/v) Tween 20 in sterile water. These sodium alginate solutions were sprayed on the leaf surface of different tomato plants until run-off point. Control plants were sprayed with 0.02% Tween 20 in sterile water. In order to facilitate maximum absorption of elicitor molecules in sodium alginate pretreated leaves, both the elicitor treated and control plants were left undisturbed for 2 days.

### Pathogen inoculation

Two days after the elicitor treatment, one set of the leaves of elicitor-treated and control plants were inoculated with *A*. *solani* as described previously [27]. Briefly, the leaves of treated plants were wounded (one wound per leaf) using a sterile needle, and a plug (5-mm diameter) of *A*. *solani* mycelium from the actively growing culture was placed on each wound. The wounded leaves of water-pretreated control plants were inoculated with droplets of sterile distilled water. Leaves were then harvested after 12, 24, 36, 40, and 60 h to determine the levels of defense-related antioxidant enzymes.

### Fluorescence microscopic studies

Histological examination of *A*. *solani*-infected tomato leaves using fluorescence microscope was performed as described by Dugyala et al. [28]. After fixing and clearing of leaves in a mixture of ethanol:chloroform (3:1) containing 0.15% (w/v) trichloroacetic acid (TCA) for 18 h, the leaves were stained with Uvitex-2B (Polysciences, Inc., USA) for 5 min. Subsequently, 2-cm sections were cut from the stained leaf samples and mounted on slides for observation under a fluorescence microscope (excitation at 493, emission at 636 nm; Leica Microscope DM 2500, Germany).

### Scanning electron microscopy (SEM) studies

SEM analysis was performed as described by Spricigo et al. [29]. Experimental leaf samples were collected on day 8 and washed thoroughly with sterile glass distilled water. The leaves were pat-dried with tissue paper, fixed in 2.5% glutaraldehyde solution with sodium cacodylate buffer (0.2 M, pH 7.2) at 4°C for 24 h, and dehydrated with acetone gradient. The samples were then frozen at −20°C and placed in a freeze-dryer under vacuum pressure at −40°C for 2 h. Subsequently, the samples were fastened with adhesive tape, coated with gold, and viewed using a scanning electron microscope (Hitachi E-1010, Japan).

### Histochemical assay of H_2_O_2_ and O_2_^−^ radicals

The level of O_2_^−^ radicals was measured using nitro blue tetrazolium chloride (NBT) staining solution as described by Kumar et al. [30]. Briefly, leaf samples were incubated in 0.05% NBT solution and placed in a standard laboratory shaker at 80 rpm for 4 h. The reaction was terminated by immersing the leaves in 95% boiling ethanol for 10 min, and the leaves were then visualized under normal light.

Qualitative analysis of H_2_O_2_ in the experimental leaves was performed using 3,3′-diaminobenzidine (DAB) staining as described by Daudi and Brien [31]. Briefly, tomato leaves were stained with 2-ml DAB solution and kept in dark for 4 h. The DAB solution was then replaced with bleaching solution [ethanol:acetic acid:glycerol (3: 1: 1)], and the leaves were incubated in that solution at 95°C for 15 min. Subsequently, the leaves were immersed in fresh bleaching solution for 30 min and the leaves were photographed.

### H_2_O_2_ assay

Quantitative analysis of H_2_O_2_ in tomato leaves was performed as described by Sellers [32]. Experimental leaves (0.2 g) were thoroughly ground in liquid nitrogen and homogenized in 500 μl of 50 mM 4-(2-hydroxyethyl)-1-piperazineethanesulfonic acid (HEPES) buffer (pH 7.4). The supernatant (100 μl) was then added to the reaction mixture containing 800 μl of 50 mM HEPES buffer (pH 7.4) and 100 μl of potassium titanium oxalate (2.5% in 20% H_2_SO_4_). The absorbance was recorded at 410 nm, and the level of H_2_O_2_ was measured from its standard curve.

### Lipid peroxidation assay

Lipid peroxidation was assayed using the method described by Heath and Pecker [33]. Briefly, 100 mg of experimental leaf sample was ground in liquid nitrogen and then homogenized in 1 ml of 5% TCA. The supernatant (1 ml) was incubated with 4 ml of 20% TCA containing 0.5% thiobarbituric acid at 95°C for 30 min. The reaction was then stopped by cooling the mixture on ice for 10 min followed by centrifugation at 6000 rpm for 15 min. The absorbance was read at 532 nm. Non-specific turbidity correction was read at 600 nm. Malondialdehyde (MDA) concentration was calculated using the extinction co-efficient of 155 mM^−1^cm^−1^. The value was expressed as μmol g^−1^ fresh weight.

### Antioxidant enzyme assay

Leaf tissue (0.2 g) was ground in liquid nitrogen using pre-chilled mortar and pestle, homogenized using 2 ml of potassium phosphate buffer (0.1 M, pH 7.5) containing 10 μl of 0.1 M disodium ethylenediaminetetraacetic acid (Na_2_-EDTA) and 1% polyvinylpyrrolidone at 4°C, and then centrifuged at 4,500 ×*g* for 30 min at 4°C. The cell-free supernatant thus obtained was used as the enzyme source for evaluation. Total protein in leaves was quantified by the dye-binding method described by Bradford using bovine serum albumin fraction V (Sigma, Bangalore) as the standard [34].

CAT activity was measured using the method described by Volk and Feierabend [35]. Briefly, 60 μl of enzyme extract was added in 3 ml of reaction mixture containing potassium phosphate buffer (0.1 M, pH 7.0) and H_2_O_2_ (14 mM). H_2_O_2_ consumption was monitored spectrophotometrically at 240 nm. The enzyme activity was then determined using the molar extinction co-efficient of 36 M^−1^cm^−1^. CAT activity was expressed in min mg^−1^ protein.

GPX activity was measured using the method described by Volk and Feierabend [35]. Briefly, 50 μl of enzyme extract was added to 3 ml of reaction mixture containing 2.93 ml of sodium phosphate buffer (0.1 M, pH 7.0), 9.8 μl of guaiacol (30 mM), and 2 μl of 6.5 mM H_2_O_2_. The increase in absorbance was read at 470 nm. GPX activity was expressed as Δ470 min^−1^ mg^−1^ protein.

For GPX isoenzyme analysis, proteins were separated on a 7% native polyacrylamide gel electrophoresis (PAGE) gel under non-reducing condition at 4°C as per the method described by Davis, and the gel was immersed in 0.018 M guaiacol at room temperature for 30 min [36]. The gel was then rinsed twice with deionized water and immersed in 1% (v/v) glacial acetic acid containing 0.015% H_2_O_2_ until the development of dark brown bands.

SOD activity was measured using the method described by Beauchamp and Fridovich based on its ability to inhibit photochemical reduction of NBT [37]. Briefly, 70 μl of leaf extract was added to 3 ml of reaction mixture containing 0.05 mM EDTA, 13 mM methionine, 75 μM NBT, and 20 μM riboflavin in potassium phosphate buffer (50 mM, pH 7.8). The blank reading was set using an enzyme extract-lacking sample incubated in light (30 μE m^−2^ s^−1^ light intensity) and another such sample incubated in dark. The absorbance was read at 560 nm after 10 min of incubation, and the dark-incubated reaction mixture for each sample was used as blank. The enzyme activity was expressed in g^−1^ fresh weight.

SOD isoforms were separated on native PAGE as described earlier, and the gel was stained in a mixture containing 10-mg NBT, 75-mg Na_2_-EDTA, and 3-mg riboflavin dissolved in 100 ml of 0.1 M Tris-HCl buffer (pH 8.2) in the dark at 25°C for 30 min. The enzyme isoforms were visualized by illuminating the gel for 10 min.

### Protease inhibitor assay

Chymotrypsin inhibitor activity was assayed following the method described by Schwert and Takenaka using tyrosine ethyl ester hydrochloride (TEE) as the substrate (Sigma and Co.) [38]. Briefly, tomato leaf extract (50 μl) was incubated with chymotrypsin (50 μl) for 5 min at room temperature. The control cuvette contained L-tyrosine instead of TEE. Trypsin inhibitor activity was determined following the method described by Schwert and Takenaka using p-tosyl arginine methyl ester hydrochloride as the substrate (Sigma and Co.) [38]. Autoclaved trypsin was used as the control. Inhibitor activity was expressed as inhibitor units per milligram of protein. An inhibitor unit was considered as the amount of inhibitor that reduces the hydrolysis of 1 mol of substrate per minute under standard conditions.

### Cell death analysis

Cell death analysis of *A*. *solani*-infected tomato leaves was performed as described by Levine et al. [39]. Sodium alginate-pretreated leaves inoculated with *A*. *solani* were collected on day 8. Leaves showing browning symptoms were collected and ground with 1 ml of sterile glass distilled water. Cell death was analyzed by staining with Evans blue dye and measuring the absorbance at 600 nm.

### Quantitative real-time polymerase chain reaction (qRT-PCR) analysis

Sodium alginate-pretreated tomato leaves inoculated with *A*. *solani* were harvested on day 8 for gene expression analysis. RNA isolation, cDNA synthesis, and expression analysis were performed as described by Sumithra Devi and Radhakrishnan [22]. Elongation factor 1 alpha was used as the internal control for normalizing qRT-PCR data, and its gene was amplified from each cDNA template using specific primers (S1 Table). The qRT-PCR data were acquired and analyzed using the Light Cycler software version 4.1.

### Statistical analysis

Experimental data were obtained from three replicates. The data are expressed as the mean ± standard error of the mean. Statistical analysis was performed using one-way analysis of variance (ANOVA) with replication and Student’s *t*-test. Duncan’s test was performed to determine the significant differences at p < 0.01 and p < 0.05. All statistical analyses were performed using the OriginPro software version 8.5 (OriginLab Corporation, Northampton, USA).

## Results

### Effect of sodium alginate pretreatment on subsequent *A*. *solani* infection in tomato plants

**Fig 1:**
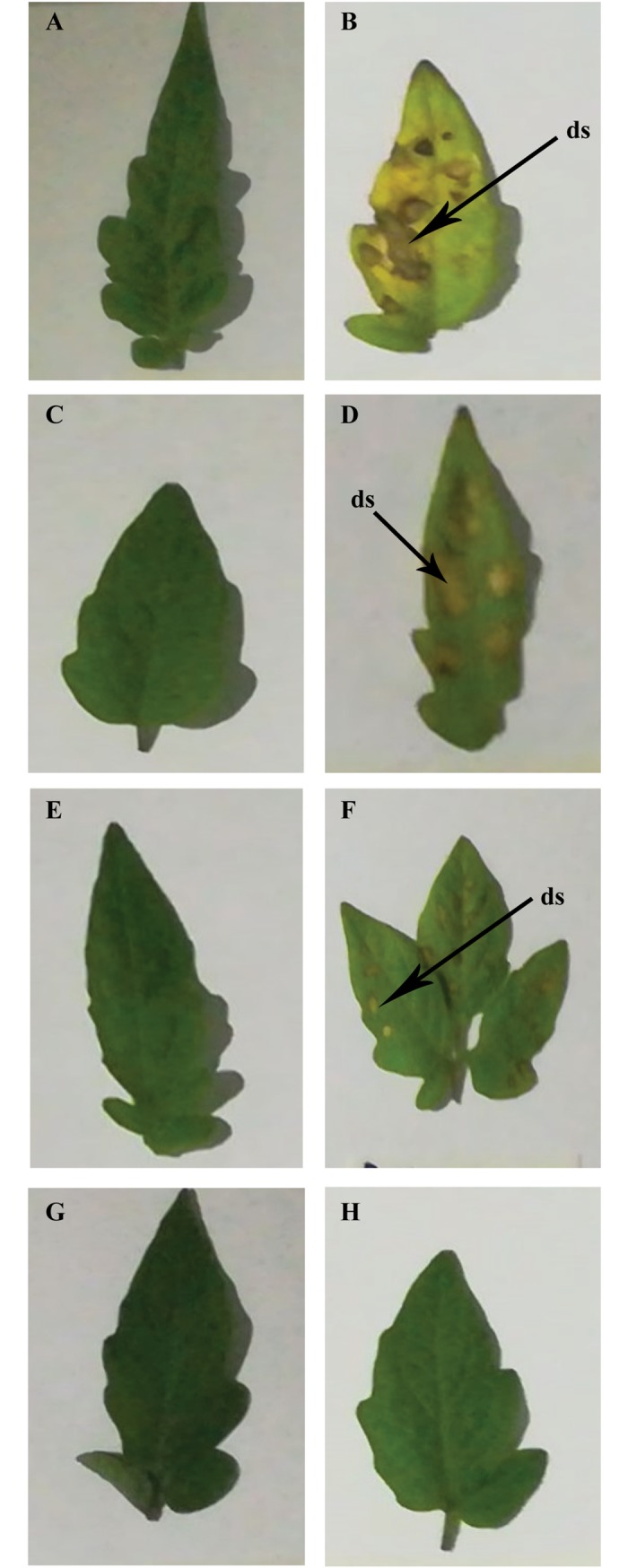
Manifestation of disease symptoms on tomato leaves on day 8 after *A*. *solani* inoculation. A: Untreated control leaves; B: Untreated *S*. *lycopersicum* leaves inoculated with *A*. *solani*; C: *S*. *lycopersicum* leaves pretreated with 0.2% sodium alginate; D: *S*. *lycopersicum* leaves pretreated with 0.2% sodium alginate and inoculated with *A*. *solani*; E: *S*. *lycopersicum* leaves pretreated with 0.4% sodium alginate; F: *S*. *lycopersicum* leaves pretreated with 0.4% sodium alginate and inoculated with *A*. *solani*; G: *S*. *lycopersicum* leaves pretreated with 0.6% sodium alginate; H: *S*. *lycopersicum* leaves pretreated with 0.6% sodium alginate and inoculated with *A*. *solani*. ds: disease symptom (early blight).

The tomato plants were pretreated by spraying different sodium alginate concentrations, i.e., 0.2%, 0.4%, and 0.6%, on their leaves, while the control plants were pretreated with water, followed by *A*. *solani* inoculation. Interestingly, sodium alginate-pretreated leaves showed significantly decreased lesion development and disease severity after *A*. *solani* infection (Fig 1A–1H). In water-pretreated control leaves inoculated with *A*. *solani*, the area of necrotic lesions and the yellowing throughout the leaves gradually increased (Fig 1B), whereas the sodium alginate-pretreated tomato leaves showed a significant dose-dependent decrease in necrotic lesion at the site of pathogen infection (Fig 1F–1H). These results suggest that the application of sodium alginate as an elicitor can effectively enhance the resistance mechanism against *A*. *solani* infection in tomato plants. The results further demonstrate that foliar spray with 0.6% sodium alginate conferred the most significant resistance against *A*. *solani* (Fig 1H).

### Sodium alginate pretreated tomato leaves show reduced *A*. *solani* infection

**Fig 2:**
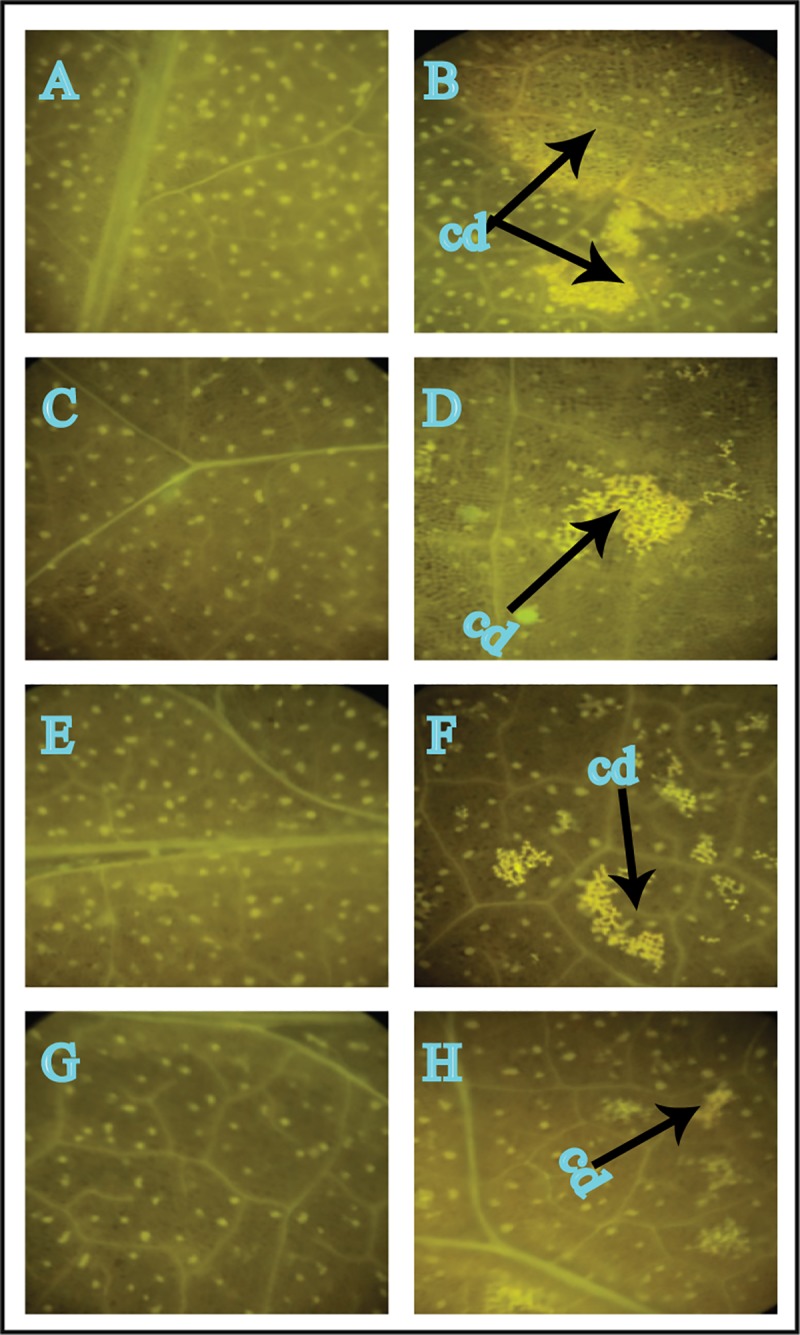
Fluorescence microscopy analysis of *A*. *solani* colonization within tomato leaves on day 8. A: Untreated control leaves; B: Untreated *S*. *lycopersicum* leaves inoculated with *A*. *solani*; C: *S*. *lycopersicum* leaves pretreated with 0.2% sodium alginate; D: *S*. *lycopersicum* leaves pretreated with 0.2% sodium alginate and inoculated with *A*. *solani*; E: *S*. *lycopersicum* leaves pretreated with 0.4% sodium alginate; F: *S*. *lycopersicum* leaves pretreated with 0.4% sodium alginate and inoculated with *A*. *solani*; G: *S*. *lycopersicum* leaves pretreated with 0.6% sodium alginate; H: *S*. *lycopersicum* leaves pretreated with 0.6% sodium alginate and inoculated with *A*. *solani*. cd: Cell death.

All of the experimental tomato leaves were assessed by fluorescence microscopy after staining with Uvitex-2B. Water-pretreated control leaves inoculated with *A*. *solani* showed symptoms of pathogen infection when compared with sodium alginate-pretreated tomato leaves inoculated with *A*. *solani* (Fig 2A–2H). Sodium alginate pretreatment reduced pathogen colonization in a dose-dependent manner (Fig 2F–2H).

### Elicitor pretreated tomato leaves demonstrate controlled fungal hyphal progression

**Fig 3:**
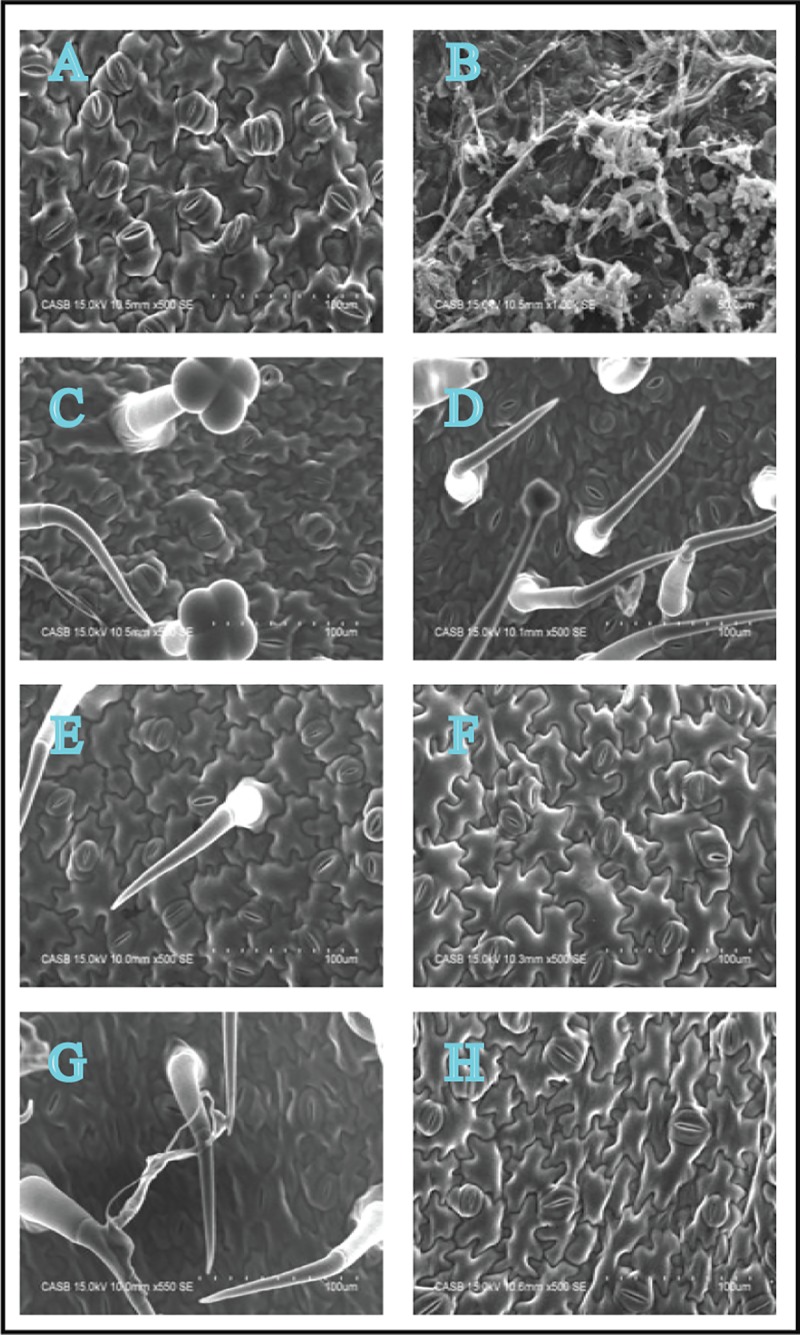
SEM analysis of *A*. *solani* infection within tomato leaves on day 8 (500× magnification). A: Untreated control leaves; B: Untreated *S*. *lycopersicum* leaves inoculated with *A*. *solani*; C: *S*. *lycopersicum* leaves pretreated with 0.2% sodium alginate; D: *S*. *lycopersicum* leaves pretreated with 0.2% sodium alginate and inoculated with *A*. *solani*; E: *S*. *lycopersicum* leaves pretreated with 0.4% sodium alginate; F: *S*. *lycopersicum* leaves pretreated with 0.4% sodium alginate and inoculated with *A*. *solani*; G: *S*. *lycopersicum* leaves pretreated with 0.6% sodium alginate; H: *S*. *lycopersicum* leaves pretreated with 0.6% sodium alginate and inoculated with *A*. *solani*. cd: Cell death.

SEM images of *A*. *solani*-infected tomato leaves are shown in Fig 3A–3H. According to the findings of SEM analysis, the infected control leaves showed a perfusion of fungal hyphae throughout the plant cells when compared with uninfected control leaves (Fig 3A and 3B). Compared with these infected control leaves, the infected leaves pretreated with sodium alginate showed a time- and concentration-dependent decrease in hyphae (Fig 3F–3H). Notably, 0.4% sodium alginate was very effective in controlling the fungal hyphal progression (Fig 3F).

### Effect of sodium alginate pretreatment on the accumulation of O_2_^−^ radicals

**Fig 4:**
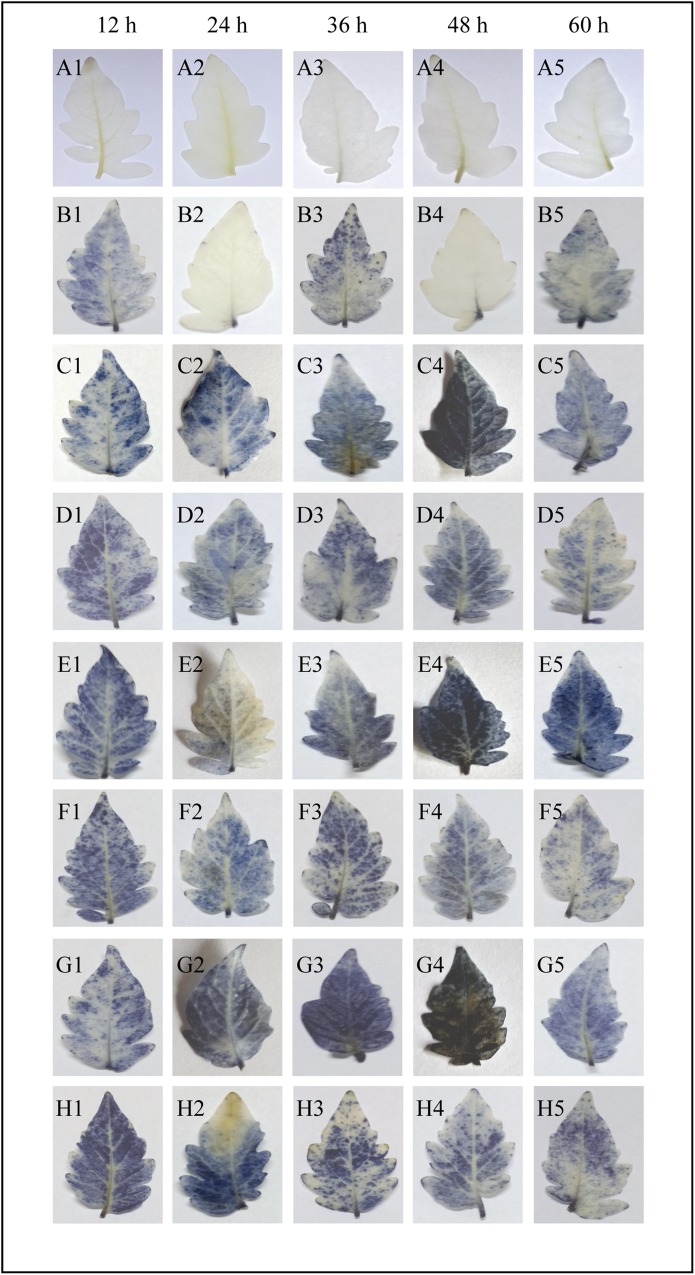
Histochemical analysis of O_2_^−^ production. The O_2_^−^ production around the site of cell death in the tomato leaves was detected by NBT staining. Compared with the control leaves (water control), sodium alginate-pretreated leaves infected with *A*. *solani* showed a time-dependent increase in the dark blue precipitation due to the reaction of NBT with O_2_^−^. A1–A5: Untreated control leaves; B1–B5: Untreated *S*. *lycopersicum* leaves inoculated with *A*. *solani*; C1–C5: *S*. *lycopersicum* leaves pretreated with 0.2% sodium alginate; D1–D5: *S*. *lycopersicum* leaves pretreated with 0.2% sodium alginate and inoculated with *A*. *solani*; E1–E5: *S*. *lycopersicum* leaves pretreated with 0.4% sodium alginate; F1–F5: *S*. *lycopersicum* leaves pretreated with 0.4% sodium alginate and inoculated with *A*. *solani*; G1–G5: *S*. *lycopersicum* leaves pretreated with 0.6% sodium alginate; H1–H5: *S*. *lycopersicum* leaves pretreated with 0.6% sodium alginate and inoculated with *A*. *solani*.

Results of the histochemical analysis of O_2_^−^ are presented in Fig 4. All of the tested sodium alginate concentrations resulted in a time- and dose-dependent accumulation of O_2_^−^ radicals in tomato leaves. Notably, 0.6% sodium alginate followed by pathogen infection resulted in a rapid accumulation of O_2_^−^ radicals during 12 to 48 h after inoculation (Fig 4H1–4H4) followed by a gradual decrease (Fig 4H5). Infected control leaves also showed a high accumulation of O_2_^−^ similar to that observed in only sodium alginate treated leaves (Fig 4B1–4B5).

### Effect of sodium alginate pretreatment on H_2_O_2_ formation

**Fig 5:**
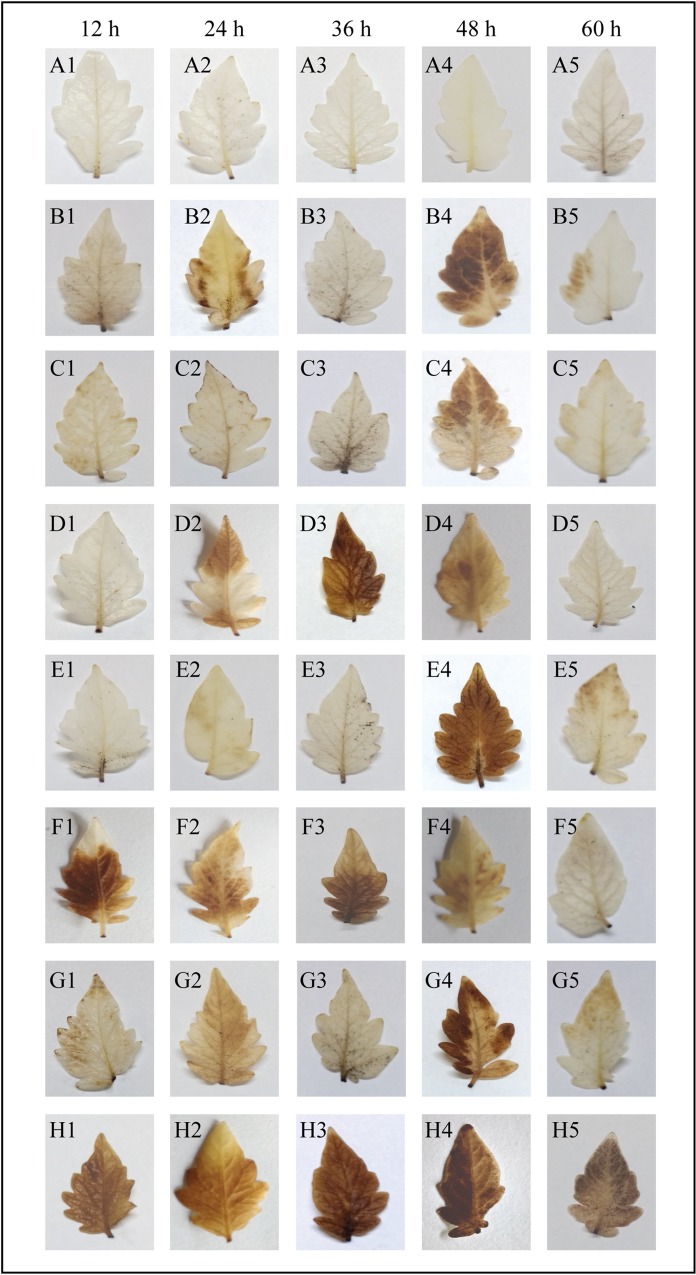
Histochemical analysis of H_2_O_2_ production. H_2_O_2_ production around the site of cell death in tomato leaves was assessed using DAB staining. Compared with the untreated control leaves, sodium alginate-pretreated leaves infected with *A*. *solani* showed a time-dependent increase in H_2_O_2_ accumulation, evident as a reddish-brown stain formed by the reaction of DAB with H_2_O_2_. A1–A5: Untreated control leaves; B1–B5: Untreated *S*. *lycopersicum* leaves inoculated with *A*. *solani*; C1–C5: *S*. *lycopersicum* leaves pretreated with 0.2% sodium alginate; D1–D5: *S*. *lycopersicum* leaves pretreated with 0.2% sodium alginate and inoculated with *A*. *solani*; E1–E5: *S*. *lycopersicum* leaves pretreated with 0.4% sodium alginate; F1–F5: *S*. *lycopersicum* leaves pretreated with 0.4% sodium alginate and inoculated with *A*. *solani*; G1–G5: *S*. *lycopersicum* leaves pretreated with 0.6% sodium alginate; H1–H5: *S*. *lycopersicum* leaves pretreated with 0.6% sodium alginate and inoculated with *A*. *solani*.

H_2_O_2_ accumulation macroscopically detected in the tomato leaves pretreated with sodium alginate and/or infected with *A*. *solani* is shown in Fig 5. Sodium alginate-pretreated leaves showed an early and significant time- and dose-dependent increase in H_2_O_2_ accumulation. In addition, 0.2% and 0.6% sodium alginate-pretreated leaves showed higher H_2_O_2_ accumulation at 48 h than water-treated control leaves (Fig 5C4 and 5H4). At 60 h after pretreatment, 0.2% and 0.6% sodium alginate-pretreated leaves showed higher H_2_O_2_ accumulation than the water-treated control leaves (Fig 5C5 and 5H5). Furthermore, a higher H_2_O_2_ accumulation was observed at 48 h in 0.2% sodium alginate-pretreated leaves infected with *A*. *solani* (Fig 5D4) when compared with only sodium alginate-treated leaves (Fig 5C4). Notably, 0.6% sodium alginate-pretreated leaves infected with *A*. *solani* showed a rapid time-dependent H_2_O_2_ accumulation 24 h onward (Fig 5H1–5H5). Pathogen-infected leaves also showed rapid H_2_O_2_ accumulation from 12 to 48 h (Fig 5B1–5B4). These results indicate that sodium alginate pretreatment followed by pathogen infection resulted in rapid H_2_O_2_ accumulation when compared with water-treated control leaves and only sodium alginate-treated leaves.

### Effect of sodium alginate pretreatment on the accumulation of H_2_O_2_ level

**Fig 6:**
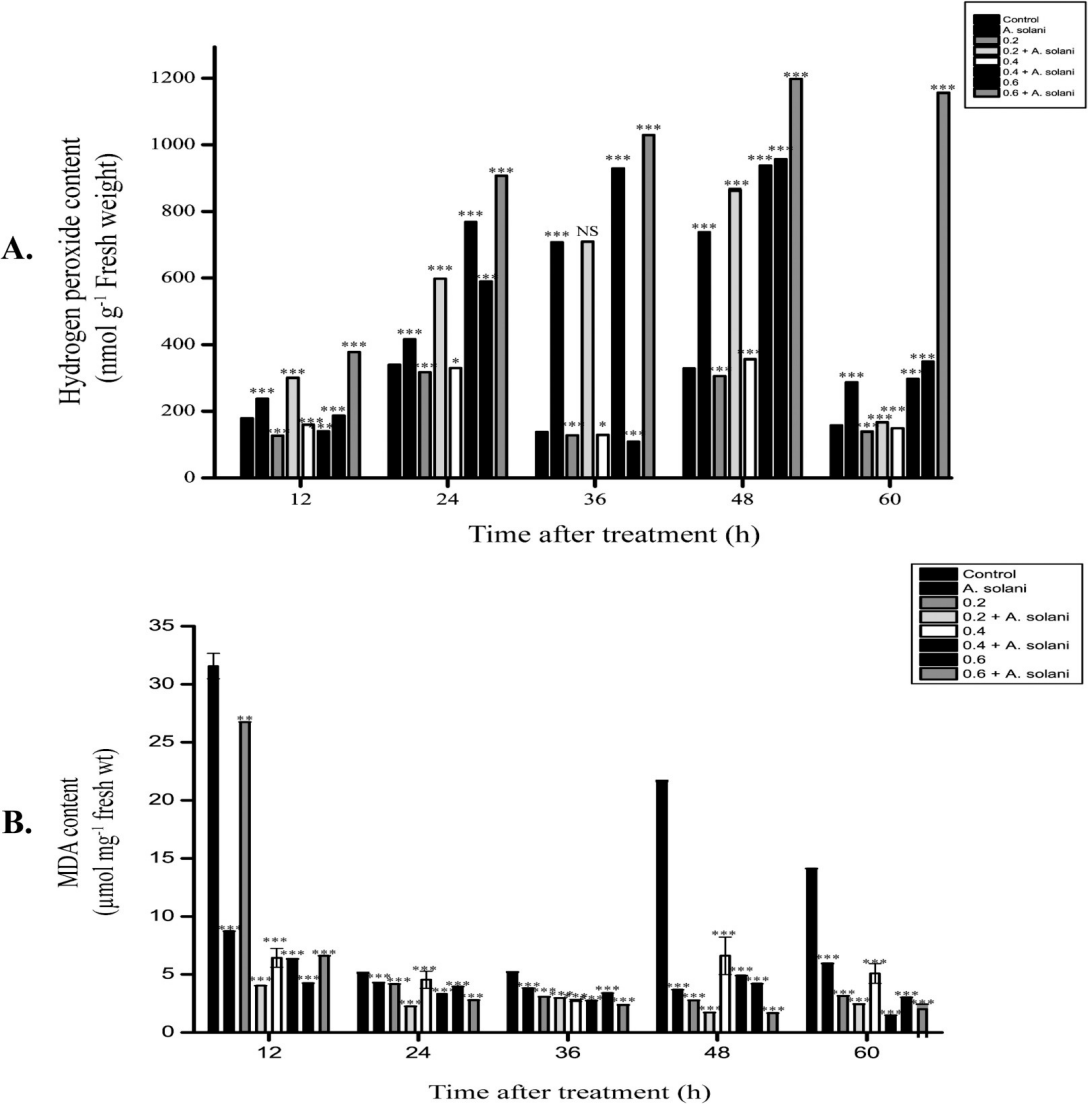
**Time course study of the levels of (A) H**_**2**_**O**_**2**_ **and (B) lipid peroxidation.** Time course study of the levels of (A) H_2_O_2_ and (B) lipid peroxidation in tomato leaves pretreated with water or sodium alginate followed by *A*. *solani* infection. The levels were measured in the leaves after the following treatments: Control, *A*. *solani* inoculation; 0.2% sodium alginate pretreatment, 0.2% sodium alginate pretreatment followed by *A*. *solani* infection; 0.4% sodium alginate pretreatment, 0.4% sodium alginate pretreatment followed by *A*. *solani* infection; 0.6% sodium alginate pretreatment, 0.6% sodium alginate pretreatment followed by *A*. *solani* infection. The values represent the mean ± standard error of the mean (n = 3). The asterisks (*, **, ***) indicate that the mean values are significantly different from those of the control at the same time point; ***P < 0.001, **P < 0.01 and *P < 0.05, ns: not significant based on Student’s *t*-test.

Notably, compared with 0.2% and 0.4% sodium alginate, 0.6% sodium alginate caused higher H_2_O_2_ accumulation at 48 h (Fig 6A). Tomato leaves pretreated with 0.2% and 0.6% sodium alginate followed by pathogen infection showed higher H_2_O_2_ accumulation at 12 h than those pretreated alone. Furthermore, leaves pretreated with 0.6% sodium alginate followed by *A*. *solani* infection showed higher H_2_O_2_ accumulation than leaves infected alone. These results indicate that tomato plants subjected to sodium alginate pretreatment followed by *A*. *solani* infection showed higher H_2_O_2_ accumulation than control, only *A*. *solani*-infected, and only elicitor-treated plants.

### Effect of sodium alginate pretreatment on the level of lipid peroxidation as a function of time

The MDA content was measured to determine the level of lipid peroxidation (Fig 6B). A high level of lipid peroxidation was observed in water-pretreated and *A*. *solani-*inoculated plants from 12 to 60 h. At 12 h, 0.2% sodium alginate-pretreated leaves showed similar level of lipid peroxidation as that shown by the control leaves (water control). However, leaves pretreated with sodium alginate (0.2%, 0.4%, and 0.6%) followed by pathogen inoculation showed a lower level of lipid peroxidation at 12 h than water-treated control leaves and only pathogen-infected leaves. The level further decreased by 30% at 48 h in tomato leaves pretreated with 0.6% sodium alginate.

### Effect of sodium alginate pretreatment on CAT activity

**Fig 7:**
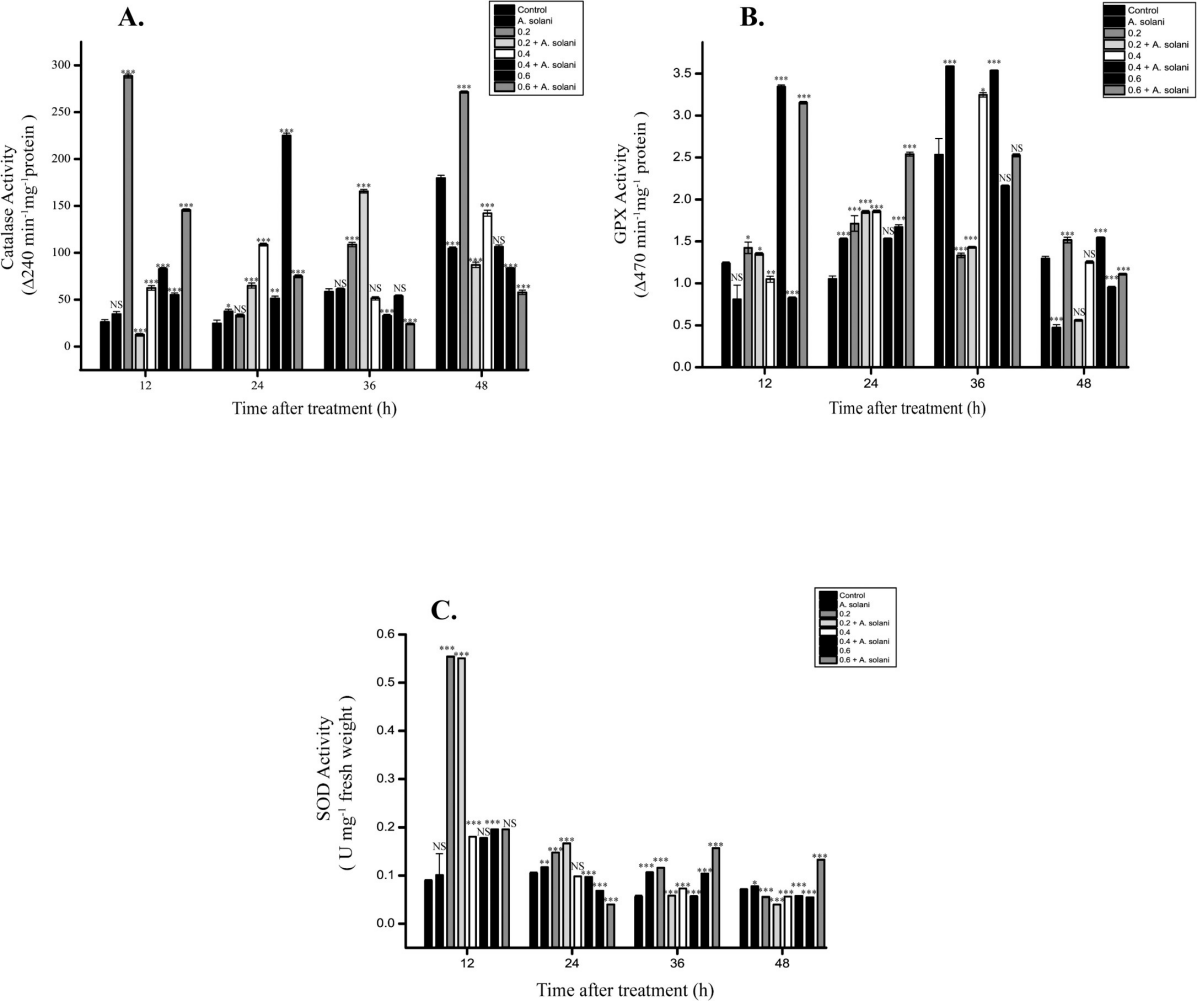
**Time course study of the levels of (A) CAT, (B) GPX, and (C) SOD.** Time course study of the levels of (A) CAT, (B) GPX, and (C) SOD in tomato leaves pretreated with sodium alginate followed by *A*. *solani* infection. The levels were measured in tomato leaves after the following treatments: Control, *A*. *solani* inoculation; 0.2% sodium alginate pretreatment, 0.2% sodium alginate pretreatment followed by *A*. *solani* infection; 0.4% sodium alginate pretreatment, 0.4% sodium alginate pretreatment followed by *A*. *solani* infection; 0.6% sodium alginate pretreatment, 0.6% sodium alginate pretreatment followed by *A*. *solani* infection The values represent the mean ± standard error of the mean (n = 3). The asterisks (*, **, ***) indicate that the mean values are significantly different from those of the control at the same time point; ***P < 0.001, **P < 0.01, and *P < 0.05, ns: not significant based on Student’s *t*-test.

The highest CAT activity was observed in tomato leaves pretreated with 0.2% sodium alginate at 12 h when compared with untreated control and only *A*. *solani*inoculated leaves. Pretreatment with all tested concentrations of sodium alginate followed by *A*. *solani* infection led to a significant increase in CAT activity at 12 h and 24 h. But, at 36 h and 48 h when compared to control, there were not much difference observed in the level of CAT activity in sodium alginate pretreated leaves followed by pathogen infection (Fig 7A).

### Effect of sodium alginate treatment on GPX activity

Results of the quantitative analysis of GPX activity are presented in Fig 7B. At 12 and 24 h, tomato leaves pretreated with sodium alginate (0.2%, 0.4%, and 0.6%) showed higher GPX activity than untreated control and only *A*. *solani*-infected leaves. A gradual decrease was observed in GPX activity from 12 to 48 h in leaves pretreated with 0.6% sodium alginate followed by *A*. *solani* infection. On the other hand, 0.4% of sodium alginate pretreated leaves followed by *A*. *solani* infection showed a transient induction of GPX from 12 h to 48 h when compared with all other treatments as well as water-pretreated control and only pathogen-infected leaves.

### Effect of sodium alginate pretreatment on SOD activity

Leaves pretreated with 0.2% sodium alginate with or without subsequent *A*. *solani* infection showed the highest SOD activity at 12 h. In contrast, leaves pretreated with 0.4% sodium alginate with or without subsequent *A*. *solani* infection showed a decrease in SOD activity from 12 to 48 h (Fig 7C). Overall, 0.6% of sodium alginate pretreatment followed by pathogen infected leaves showed a gradual but slight increase in the SOD level from 12 h to 48 h when compared with uninfected control and infected control leaves. On the other hand, SOD activity was found to be decreased gradually in case of 0.2% and 0.4% of sodium alginate pretreatment followed by pathogen infected leaves.

### Effect of sodium alginate pretreatment on GPX activity

**Fig 8:**
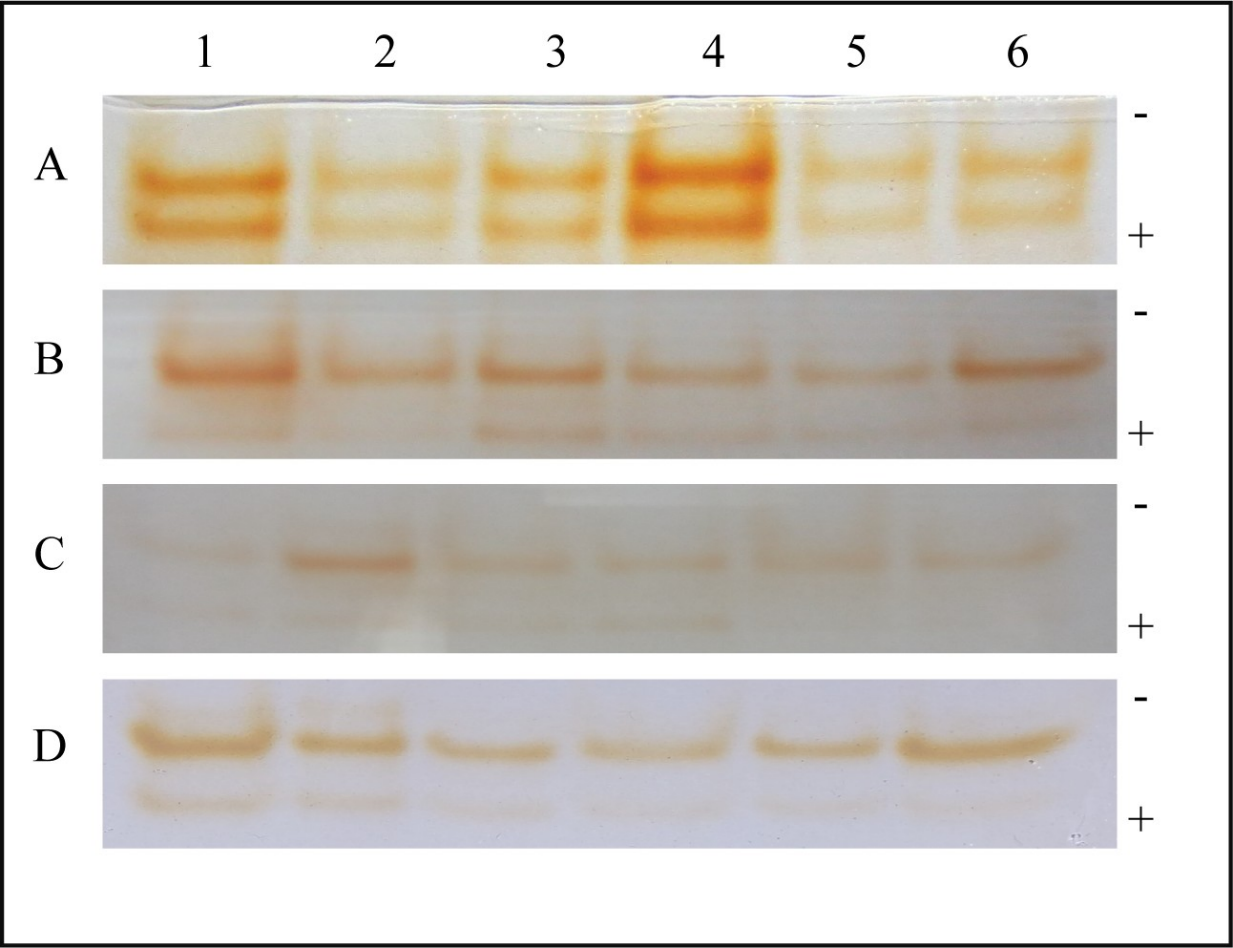
GPX activity revealed by guaiacol and H_2_O_2_ staining in sodium alginate-pretreated tomato leaves infected with *A*. *solani*. Each lane contains 40 μg of enzyme sample extracted from tomato leaves harvested at different time points after the following treatments: Lane 1: Control; Lane 2: *A*. *solani* inoculation; Lane 3: 0.4% sodium alginate pretreatment; Lane 4: 0.4% sodium alginate pretreatment followed by *A*. *solani* inoculation; Lane 5: 0.6% sodium alginate pretreatment; Lane 6: 0.6% sodium alginate pretreatment followed by *A*. *solani* infection. A: 12 h; B: 24 h; C: 36 h; D: 48 h. The (-) and (+) signs indicate the direction of migration of enzyme samples.

GPX in sodium alginate-pretreated tomato leaves inoculated with *A*. *solani* was analyzed qualitatively by native PAGE followed by staining with guaiacol and H_2_O_2_ (Fig 8 and S1 Fig). At 12 h, although, there was no evidence of formation of new isoform, but as compared to control, a change in intensity of GPX activity was observed in 0.4% sodium alginate-pretreated tomato leaves inoculated with *A*. *solani*. A time dependent decrease in GPX activity was observed from 12 h to 48 h in all the samples pretreated with sodium alginate followed by pathogen infection as compared to control.

### Native-PAGE analysis of SOD isoforms

**Fig 9:**
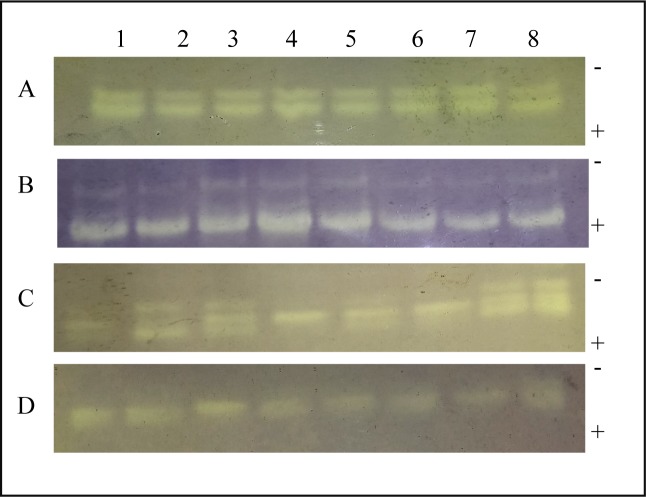
SOD activity revealed by NBT and riboflavin staining in sodium alginate-pretreated tomato leaves infected with *A*. *solani*. Each lane contains 40 μg of enzyme sample extracted from tomato leaves harvested at different time points after the following treatments: Lane 1: Control; Lane 2: *A*. *solani* inoculation; Lane 3: 0.2% sodium alginate pretreatment; Lane 4: 0.2% sodium alginate pretreatment followed by *A*. *solani* inoculation; Lane 5: 0.4% sodium alginate pretreatment; Lane 6: 0.4% sodium alginate pretreatment followed by *A*. *solani* infection; Lane 7: 0.6% sodium alginate pretreatment; Lane 8: 0.6% sodium alginate pretreatment followed by *A*. *solani* infection. A: 12 h; B: 24 h; C: 36 h; D: 48 h. The (-) and (+) signs indicate the direction of migration of enzyme samples.

Qualitative analysis of SOD in sodium alginate-pretreated tomato leaves inoculated with *A*. *solani* was performed by native PAGE, followed by staining with NBT and riboflavin. The gel analysis showed two bands at 12 h, three bands at 24 and 36 h and one band at 48 h respectively in order of increasing migration. The bands were present in uninfected control, infected control and sodium alginate pretreated leaves with or without infection. The intensity of bands decreased in a time dependent manner for all the samples (Fig 9 and S2 Fig).

### Effect of sodium alginate treatment on Trypsin inhibitor activity

**Fig 10:**
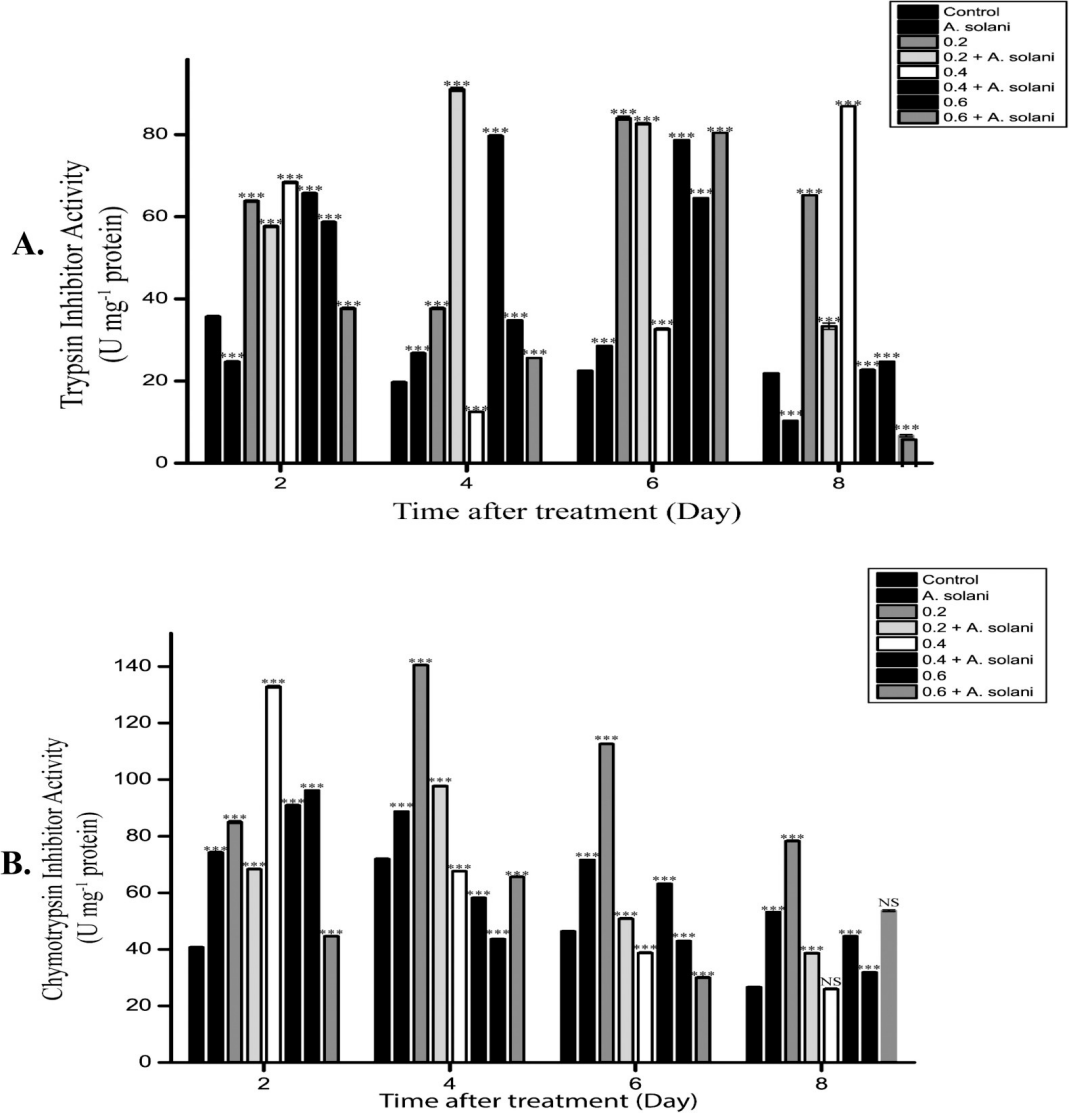
**Changes in A) trypsin and B) chymotrypsin inhibitory activities.** Changes in A) trypsin and B) chymotrypsin inhibitor activities in tomato leaves harvested at different time points after the following treatments: Control, *A*. *solani* inoculation, 0.2% sodium alginate pretreatment, 0.2% sodium alginate pretreatment followed by *A*. *solani* infection; 0.4% sodium alginate pretreatment, 0.4% sodium alginate pretreatment followed by *A*. *solani* infection; 0.6% sodium alginate pretreatment, 0.6% sodium alginate pretreatment followed by *A*. *solani* infection The values represent the mean ± standard error of the mean (n = 3). The asterisks (*, **, ***) indicate that the mean values are significantly different from those of the control at the same time point; ***P < 0.001, **P < 0.01, and *P < 0.05, ns: not significant based on Student’s *t*-test.

Sodium alginate-pretreated tomato leaves showed increased trypsin inhibitor activity when compared with water-treated control leaves. Similarly, *A*. *solani*-infected leaves showed increased trypsin inhibitor activity when compared with uninfected control leaves. However, a significant increase in trypsin inhibitor activity was observed from day 2 onward in sodium alginate-pretreated tomato leaves infected with *A*. *solani* (Fig 10A) when compared with pathogen alone infected leaves. Further, an increase in trypsin inhibitor activity was observed in 0.2% sodium alginate-pretreated leaves infected with *A*. *solani* when compared with only pretreated leaves. Notably, 0.2% sodium alginate followed by pathogen infection resulted in 2- and 3-fold increase in trypsin inhibitor activity on day 6 when compared with 0.4% and 0.6% sodium alginate pretreatment, respectively. Overall, these results indicate that sodium alginate pretreatment followed by *A*. *solani* infection led to a significant increase in trypsin inhibitor activity.

### Effect of sodium alginate on Chymotrypsin inhibitor activity

Compared with untreated control leaves, sodium alginate-pretreated tomato leaves showed a dose-dependent increase in chymotrypsin inhibitor activity (Fig 10B). Similarly, *A*. *solani*-infected leaves also showed increased chymotrypsin inhibitor activity when compared with uninfected control leaves. Chymotrypsin inhibitor activity decreased after day 2 in sodium alginate-pretreated leaves inoculated with *A*. *solani* infection. Leaves pretreated with 0.2% sodium alginate alone showed increased chymotrypsin inhibitor activity when compared with untreated control. Notably, when compared to untreated control leaves, 0.6% sodium alginate-pretreated leaves showed decreased activity on day 4 and 6. In addition, compared with tomato leaves pretreated with 0.4% and 0.6% sodium alginate, those pretreated with 0.2% sodium alginate showed maximum chymotrypsin inhibitor activity on all days.

### Analysis of the effect of sodium alginate in reducing the cell death of tomato leaves caused by *A*. *solani*

**Fig 11:**
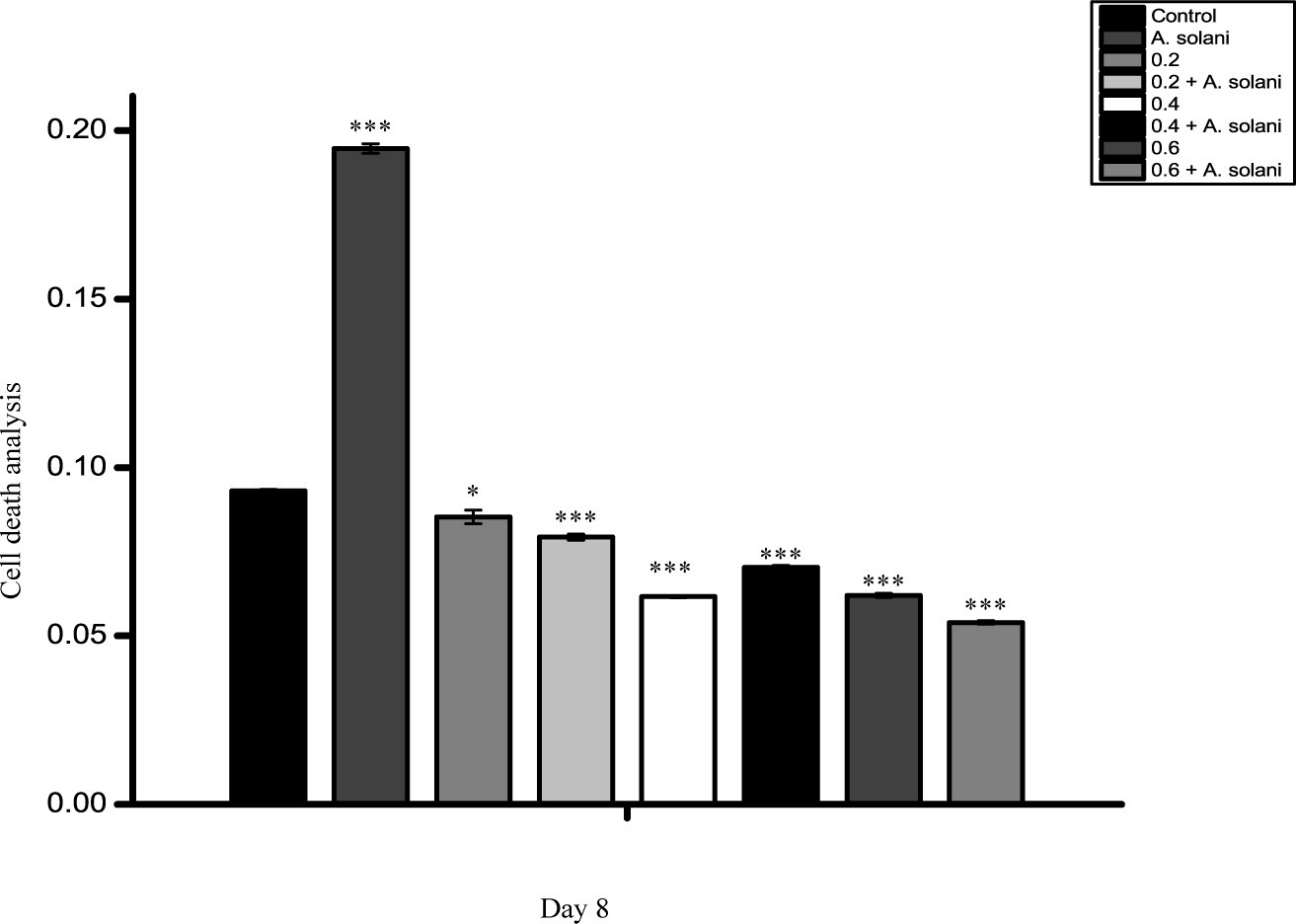
Analysis of cell death in tomato leaves. The tomato leaves for cell death analysis were harvested on day 8 after the following treatments: Control, *A*. *solani* inoculation; 0.2% sodium alginate pretreatment, 0.2% sodium alginate pretreatment followed by *A*. *solani* infection; 0.4% sodium alginate pretreatment, 0.4% sodium alginate pretreatment followed by *A*. *solani* infection; 0.6% sodium alginate pretreatment, 0.6% sodium alginate pretreatment followed by *A*. *solani* infection. The values represent the mean ± standard error of the mean (n = 3). The asterisks (*, **, ***) indicate that the mean values are significantly different from those of the control at the same time point; ***P < 0.001, **P < 0.01, and *P < 0.05, ns: not significant based on Student’s *t*-test.

Cell death caused by *A*. *solani* infection was investigated using Evans blue dye (Fig 11). The first appearance of browning was observed on day 2, and it progressed until day 10 in infected leaves when compared with uninfected leaves. The highest cell death was observed in non-treated leaves infected with *A*. *solani*. Sodium alginate-pretreated leaves showed a 2-fold decrease in cell death when compared with pathogen-infected leaves, in which cell death was equivalent to that in untreated control plants. A dose-dependent decrease in cell death was observed in sodium alginate-pretreated tomato plants infected with *A*. *solani*.

### Effect of sodium alginate pretreatment on the induction of plant defense gene expression

**Fig 12:**
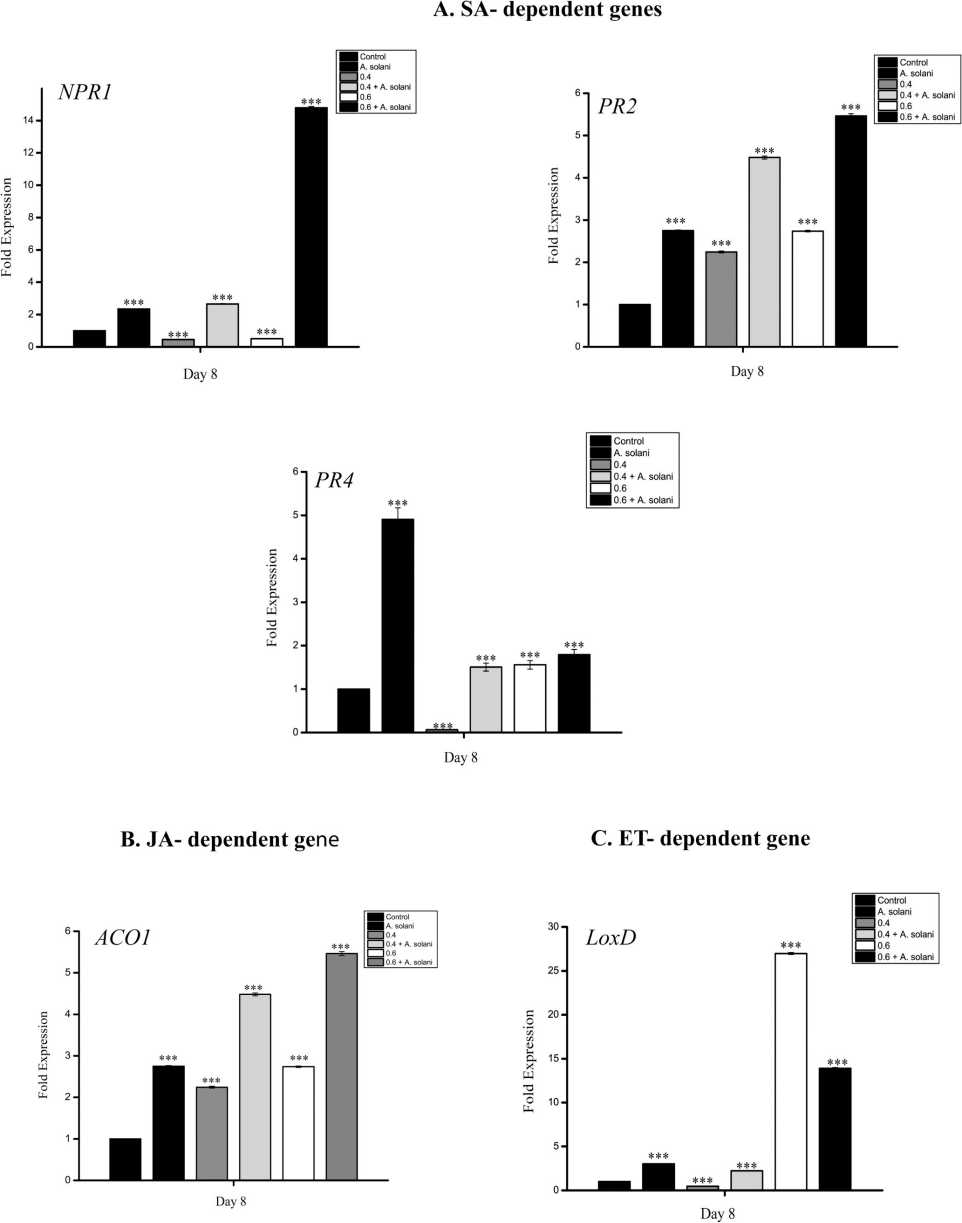
**Relative expression of A) SA-dependent, B) JA-dependent, and C) ET-dependent defense genes.** Relative expression of A) SA-dependent, B) JA-dependent, and C) ET-dependent defense genes in tomato leaves harvested on day 8 after the following treatments: Control, *A*. *solani* inoculation; 0.4% sodium alginate pretreatment, 0.4% sodium alginate pretreatment followed by *A*. *solani* infection; 0.6% sodium alginate pretreatment, 0.6% sodium alginate pretreatment followed by *A*. *solani* inoculation. The relative gene expressions of SA-dependent *NPR1*, *PR2*, and *PR4* transcripts; JA-dependent *LoxD* transcript; and ET-dependent *ACO1* transcript were calculated using the comparative Ct method. One-way ANOVA was used to determine whether the sample means differed significantly at ***P < 0.001, **P < 0.01, or *P < 0.05. The error bars represent the 95% confidence interval calculated from ANOVA. The values represent means (of three replicates) ± standard error of the mean.

To understand the mechanism underlying sodium alginate-induced resistance in tomato, the expression of five plant defense marker genes, namely *PR2 (β-1*,*3-Glucanase)*, *PR4 (Chitinase)*, *NPR1 (Non-expressor of pathogenesis related protein 1)* belonging to the SA-dependent pathway, *ACO1* belonging to the ET-dependent pathway, and *LoxD (Lipoxygenase D)* belonging to the JA-dependent pathway, was evaluated (Fig 12).

In this study, *PR2* expression was significantly upregulated by 2.5-fold in *A*. *solani*-infected tomato leaves compared to water treated control plants (Fig 12A). In particular, 0.4% and 0.6% sodium alginate-pretreated leaves infected with *A*. *solani* showed 4- and 5-fold increase in *PR2* expression, respectively, compared with untreated, only pathogen-infected control leaves. Only sodium alginate-pretreated leaves showed almost 2-fold increase in *PR2* expression when compared with untreated control leaves and a decrease when compared with only pathogen-infected leaves. *PR4* expression was upregulated in only pathogen-infected leaves when compared with uninfected control leaves and 0.4% and 0.6% sodium alginate-pretreated leaves infected with pathogen (Fig 12A). *NPR1* expression was upregulated in sodium alginate-pretreated and pathogen-infected leaves on day 8, with a 7-fold increase observed in 0.6% sodium alginate-pretreated leaves infected with pathogen when compared with the expression in untreated control leaves and only pathogen-infected leaves (Fig 12A). A similar trend was also observed in *Lox D* expression. Notably, only 0.6% sodium alginate-pretreated leaves and sodium alginate-pretreated leaves infected with pathogen showed a 20- and 5-fold increase in *Lox D* expression, respectively, compared with control leaves, which showed higher *Lox D* expression than that in only pathogen-infected leaves (Fig 12B). *ACO1* expression was significantly upregulated by 6- and 3-fold in only sodium alginate-pretreated leaves and sodium alginate-pretreated leaves infected with *A*. *solani*, respectively, compared with control leaves and only pathogen-infected leaves (Fig 12C).

## Discussion

Polysaccharides derived from algae have shown multidirectional stimulatory effects in plants [40]. Among such polysaccharides, sodium alginate derived mainly from brown algae (class Phaeophyceae) can stimulate seed germination [25], plant growth and development [41], ion uptake and transport [42], and photosynthesis [43]. It can also increase pigment [44], protein [45], and secondary metabolite [43] assimilation. Sodium alginate also inhibits some plant diseases [46]. In the present study, we found that sodium alginate pretreatment can induce resistance against necrotrophic fungal pathogen *A*. *solani* in tomato seedlings. The results of fluorescence microscopy and SEM indicated that foliar spray of sodium alginate (0.6%) used as a pretreatment remarkably prevented pathogen growth and reduced disease severity.

O_2_^−^ is the primary ROS produced in plant cells, and it initiates a cascade of reactions to generate other ROS in a time- and space-dependent manner [47]. O_2_^−^ and H_2_O_2_ generated during early stages of *A*. *solani* colonization are immediately quenched at later time points by the efficient antioxidative system, thus protecting plant cells from oxidative damages. O_2_^−^ is moderately reactive and doesn’t cause severe cell damage due to its short half-life. On the other hand, H_2_O_2_ is present in plant cells under normal as well as stressed conditions and has positive as well as negative effects on plant health. At low concentration, it acts as a regulatory signal for various physiological processes but at high concentration, it damages the plant cell [48]. In our histochemical analysis, it has been observed that compared to O_2_^-^, the accumulation of H_2_O_2_ was higher in tomato leaves treated with 0.6% of sodium alginate followed by pathogen infection which indicates that O_2_^-^ has been immediately dismutated to H_2_O_2_ after production.

The lipid peroxidation level is an effective marker to assess the cell membrane damage caused by ROS in stressed conditions. The formation of MDA, which damages cell membrane, is a key indicator of peroxidation. Lipid peroxide production has been found to be induced by several pathogens [49]. In our experimental study, a significantly high lipid peroxidation level was observed in water-pretreated and pathogen-infected plants, whereas very low level was observed in sodium alginate-pretreated plants with or without pathogen infection. This finding indicates a correlation between ROS accumulation and lipid peroxidation level during plant-pathogen interaction.

CAT scavenges H_2_O_2_ generated in the peroxisome—the major site of H_2_O_2_ production. Environmental stress can modulate CAT activity by enhancing or suppressing it in a time-, dose- and stress-dependent manner [50]. It has also been reported that during plant-pathogen interaction, in a resistant plant, decreased catalase activity allows higher accumulation of hydrogen peroxide which prevents further pathogen infection by strengthening the cell wall, activating defense gene and hypersensitive cell death [6][51][52]. In our study, we observed that pretreatment with all tested concentrations of sodium alginate followed by *A*. *solani* infection showed less CAT activity as compared to the generation of H_2_O_2_. Thus, based on the result we can hypothesize that sodium alginate pretreatment aid in conferring disease resistance to tomato plants against *A*. *solani* by decreasing CAT activity and accumulating higher level of H_2_O_2_ at the site of infection.

GPX is widely present in animals, plants, and microbes. Under stressed conditions, GPX effectively quenches the reactive intermediate forms of O_2_^−^ and peroxy radicals [53]. In this study we observed that there was an early induction of GPX activity in the sodium alginate pretreated leaves with pathogen infection which was suppressed at the later stage. The root peroxidases of *Allium porrum*, Zea mays and *Phaseolus vulgari*s have also been reported to show the same pattern of peroxidase expression upon pathogen infection[54][55][56]. Based on these observations, it can be suggested that sodium alginate pretreatment triggers early induction of GPX in tomato plant which helps in prior onset of defense responses against pathogen infection and this existing active mechanism in turn may acts as an inhibitory signal for GPX activity at a later stage.

Since, various GPX isoforms have been reported to be present in plants, so to determine the no. of GPX isoforms in the leaves of tomato and their expression in response to pathogen infection we used the native PAGE method[57]. It was observed that two bands were present in all the samples irrespective of sodium alginate pretreatment and *A*. *solani* infection. A time dependent decrease in quantity of the expressed bands of GPX was also observed in all the sodium alginate pretreated leaves followed by pathogen infection as compared to control. Thus, we can correlate this result with the quantitative analysis of the GPX activity. Further studies will be required to identify these isoforms of GPX and their role in plant defense mechanism.

SOD plays a key role in inducing defense against oxidative stress in all aerobic organisms [58]. It catalyzes the dismutation of O_2_^−^ to O_2_ and H_2_O_2_. SOD is often correlated with increased tolerance of the plant against environmental stresses. Our study showed a correlation between increased SOD activity and increased H_2_O_2_ accumulation in 0.6% sodium alginate-pretreated plants at early stages of interaction with the pathogen when compared with water-pretreated control and only pathogen-infected plants except at 24 h. Thus, it can be suggested that higher concentration of sodium alginate pretreatment may confer resistance to tomato plants against *A*. *solani* infection by elevating the level of SOD. Further, native PAGE analysis followed by in gel staining activity revealed differential expression of three new SOD isoenzymes at 24 h and 36 h in all the samples irrespective of sodium alginate pretreatment and *A*. *solani* infection. But further investigations are required to distinguish these different isoenzymes and the role they play in defense mechanism of plants.

Protease inhibitors have been found to exhibit antifungal activity [59] probably due to the counter defense mechanism occurring during the interaction between protease and protease inhibitors of the host and pathogen [60]. In the present study, sodium alginate pretreatment of tomato leaves resulted in increased trypsin activity and decreased chymotrypsin inhibitor activities. In particular, trypsin inhibitor activity was much higher than chymotrypsin inhibitor activity during infection. This difference may be due to the different regulatory mechanisms involved in the induction of these two classes of inhibitors in tomato leaves [61]. Interestingly, the reduction in disease symptoms in terms of cell death has been reported to be correlated with increased activities of proteases, protease inhibitors, and POXs. Such increase in enzyme activity in sodium alginate-pretreated leaves in our study could have restricted the development of disease symptoms by inhibiting the growth of the pathogen *A*. *solani*. One similar study demonstrated that enhanced activities of both POX and protease inhibitors contribute to the plant defense response against *Pythium aphanidermatum* in turmeric plants [62].

Lastly, we evaluated the ability of sodium alginate to induce the expression of some defense-related genes against *A*. *solani* infection in tomato seedlings. It is known that plants are equipped with various defense genes, the expression of which is latent in healthy conditions. The induction of these defense genes in plants by a prior application of any inducer is known as induced resistance [63]. *NPR1* is the master regulator of SA-mediated SAR in plants [64]. Plant β-1,3-glucanases are a group of PR proteins belonging to the PR-2 family, whereas chitinases belong to the PR-4 family. The major cell wall components of many phytopathogenic fungi, including *A*. *solani*, are chitin and glucan. Therefore, it is believed that both β-1,3-glucanases and chitinases play an antifungal role by hydrolyzing the fungal cell wall, which in turn disintegrates the fungal cell. In addition, β-1,3-glucanases and chitinases have been shown to exhibit indirect effects via the formation of oligosaccharide elicitors, which further induce the expression of other PR proteins [65]. *LoxD* gene expression is regulated by different effectors, such as JA and abscisic acid, and also by different forms of stress, such as wounding, water deficiency, or pathogen attack [66]. Our analysis of the mRNA expression of five defense genes (*NPR1*, *PR2*, *PR4*, *LOXD*, and *ACO1*) belonging to SA-, JA-, and ET-dependent pathways in tomato leaves following sodium alginate pretreatment revealed that the expression of *NPR1*, *PR2*, *LOXD*, and *ACO1* rapidly and significantly increased and that of *PR4* decreased in 0.6% sodium alginate-pretreated tomato seedling infected with pathogen when compared with those in only pathogen-infected plants, which showed significantly upregulated *PR4* expression. Overall, our results suggest that sodium alginate induces defense responses by activating antioxidant enzymes and PR proteins against *A*. *solani*. This finding supports the application of sodium alginate in suppressing disease development in tomato seedlings.

## Conclusions

The present study demonstrated that the biopolymer sodium alginate promotes antioxidant defense and antifungal PR protein expression in tomato plants, thereby inducing resistance against *A*. *solani*-caused early blight disease. Interestingly, time-dependent oxidative burst along with the induction of SA-, JA-, and ET-responsive PR gene expression by sodium alginate suggests its efficacy in promoting plant defense. Further studies are warranted to clarify the mechanism underlying the sodium alginate-induced signal transduction pathway.

## Supporting information

S1 Table Primers used for quantitative RT-PCR. Primers are shown in the 5′-3′ orientation. (F): Forward; (R): reverse primer.
(PDF)

S1 Fig Enzyme activity staining of GPX of sodium alginate pretreated tomato leaves infected with *A*. *solani*. A) 12 h; B) 24 h; C) 36 h; D) 48 h.
(PDF)

S2 Fig Enzyme activity staining of SOD of sodium alginate pretreated tomato leaves infected with *A*. *solani*. A) 12 h; B) 24 h; C) 36 h; D) 48 h.
(PDF)
