# Supplementary material for: Sodium alginate potentiates antioxidant defense and PR proteins against early blight disease caused by Alternaria solani in Solanum lycopersicum Linn
Source: PLoS One. 2019 Sep 30;14(9):e0223216. doi: 10.1371/journal.pone.0223216 (PMC6768480; doi:10.1371/journal.pone.0223216)
Supplement: S1 Table — Primers are shown in the 5′-3′ orientation. (F): Forward; (R): reverse primer. (PDF) [file pone.0223216.s001.pdf]

S1 Table. Primers used for Quantitative RT-PCR. Primers are shown in the 5′-3′ orientation. (F): Forward; (R): reverse primer.

Gene	Primer Name	Primer Sequence (5´-3´)
NPR1	NPR1-F	GGCTAGCATGAGGAAGAAGATAG
	NPR1-R	GCCCTAAGCCGATTCAAGT
β-1,3-G	β-1,3-G-F	CCAAAGAAACCAGGAAGGACTA
	β-1,3-G-R	GCCTCTGGTCAGGTTTAAAGA
Chi 3	Chi 3-F	GTTCTGGATGACAGAACAGGAT
	Chi 3-R	ACCGTACCCTGGAACTCTATTA
ACO1	ACO1-F	CCTCAAAGACGAGCAATGGA
	ACO1-R	CACACTCTTGTACTTCCCGTTAG
LoxD	Lox D-F	CCGGAGATGCAGAGATTGTT
	Lox D-R	CACCAGCACCACACCTATT
EF	EF-F	GTCAGGTATCCTTTCCCAGAAC
	EF-R	GATGAACCTAGGCACCTCAAA
