# Supplementary material for: Sodium alginate potentiates antioxidant defense and PR proteins against early blight disease caused by Alternaria solani in Solanum lycopersicum Linn
Source: PLoS One. 2019 Sep 30;14(9):e0223216. doi: 10.1371/journal.pone.0223216 (PMC6768480; doi:10.1371/journal.pone.0223216)
Supplement: S1 Fig — A) 12 h; B) 24 h; C) 36 h; D) 48 h. (PDF) [file pone.0223216.s002.pdf]

S1 Fig. Enzyme activity staining of GPX of sodium alginate pretreated tomato leaves infected with *A. solani*.

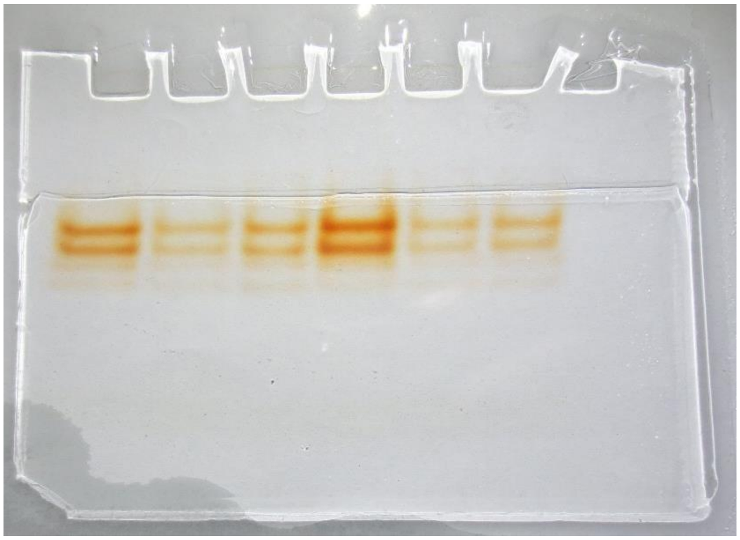

(A) 12 h

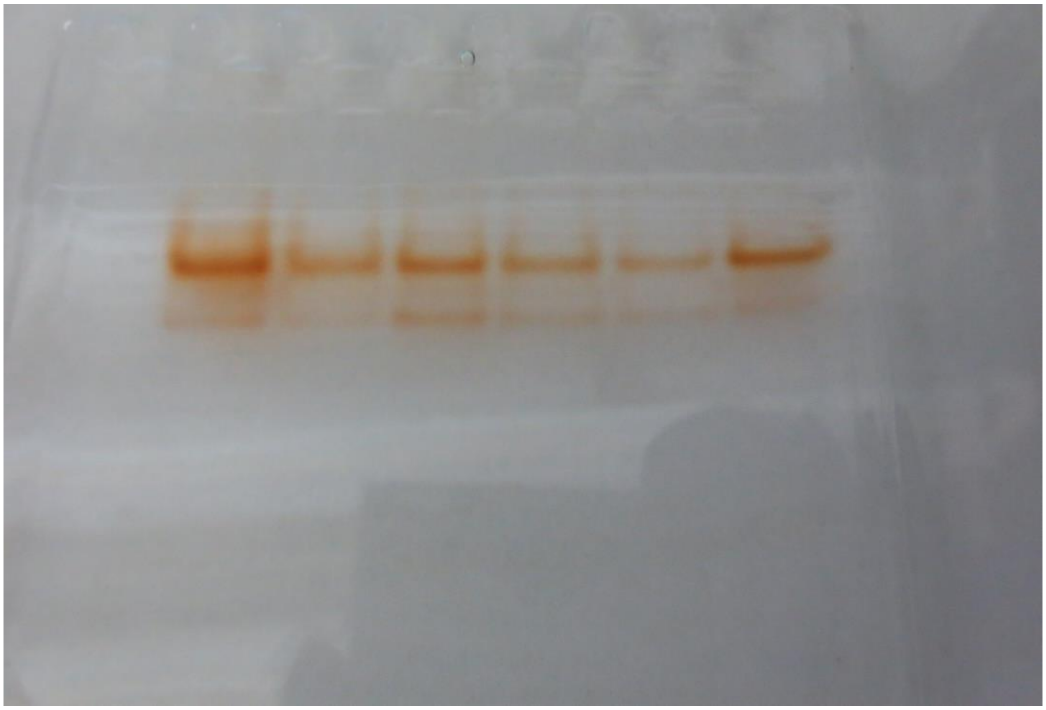

(B) 24 h

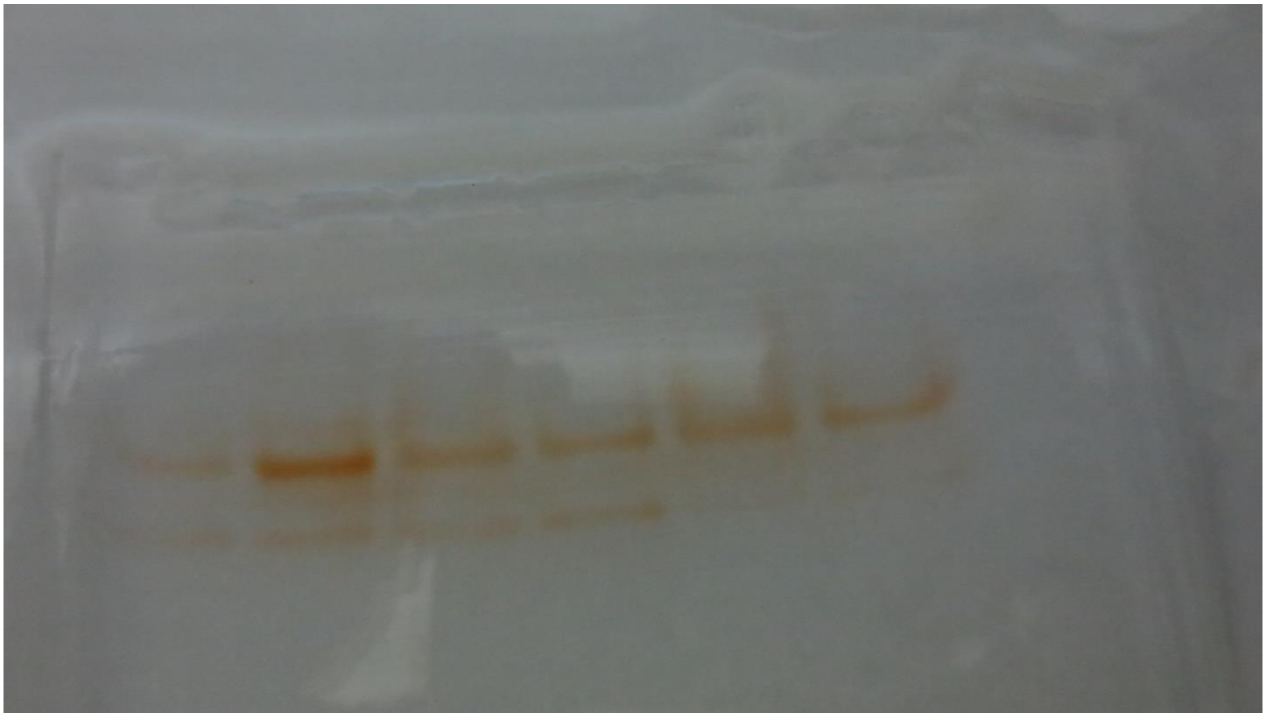

(C) 36 h
